# Lipidomics Techniques Revealed the Adipogenic Differentiation Mechanism of Bovine Adipose-Derived Neural Crest Stem Cells

**DOI:** 10.3390/ani15213191

**Published:** 2025-11-02

**Authors:** Kai Zhang, Zhaotong Liang, Yilin Ding, Xianyi Song, Rui Zhao, Yibo Yan, Xiaopeng Tang

**Affiliations:** 1 College of Animal Science, Shanxi Agricultural University, Taiyuan 030032, China; zhangkai2255@163.com (K.Z.); l3360607257@163.com (Z.L.); striverpanpan116@163.com (Y.D.); zr73498896@163.com (R.Z.); 206434yyb@sxau.edu.cn (Y.Y.); 2 State Engineering Technology Institute for Karst Desertfication Control, School of Karst Science, Guizhou Normal University, Guiyang 550025, China

**Keywords:** bovine adipose-derived neural crest stem cells, adipogenic differentiation, lipidomics, UHPLC-MS/MS, triacylglycerols, membrane lipid remodeling

## Abstract

**Simple Summary:**

Bovine adipose-derived neural crest stem cells (baNCSCs) are a unique model for studying fat formation. Using advanced lipidomics technology, we analyzed the complete changes in lipid molecules during the differentiation of baNCSCs into fat cells. We found that the transformation of these stem cells into mature fat cells involves a massive accumulation of storage fats like triglycerides (TG), as well as a sophisticated reprogramming of membrane fats and signaling lipids. This study provides a comprehensive lipid-based perspective on how fat cells develop in bovines, offering new insights for animal science and metabolic research.

**Abstract:**

Bovine adipose-derived neural crest stem cells (baNCSCs) are an ideal model for studying the mechanism of adipogenesis. Lipidomics provides a powerful technical means to comprehensively analyze the dynamic changes in lipid metabolism during cell differentiation. However, the lipidomic remodeling throughout the adipogenic differentiation of baNCSCs is still lacking in-depth research. This study used ultra-high-performance liquid chromatography–tandem mass spectrometry (UHPLC-MS/MS) to perform non-targeted lipidomic analysis on baNCSCs on day 0 (CON0) and day 9 (DIF9) of adipogenic induction and differentiation. Differential lipid metabolites were screened through multivariate statistical analysis and univariate analysis. A total of 1639 lipid molecules were identified. Compared with the CON0 group, 568 lipids were significantly altered in the DIF9 group, involving 6 major categories and 20 subclasses. The relative content and types of triacylglycerols (TAG) and diacylglycerols (DAG) increased significantly, becoming the most important markers of successful differentiation. Glycerophospholipids (GP) underwent complex remodeling, with subclasses such as phosphatidylethanolamine (PE), phosphatidylserine (PS), and cardiolipin (CL) significantly increased, indicating extensive restructuring of the cell and organelle membranes to adapt to lipid storage and energy metabolism. Sphingolipids (SP) such as ceramides (Cer) and sphingomyelins (SM) were generally downregulated. The content of acylcarnitines (ACar) and hydroxy fatty acids (FAHFA) increased, suggesting enhanced fatty acid β-oxidation and metabolic health. This study systematically reveals the comprehensive lipidome reprogramming during the adipogenic differentiation of baNCSCs, which involves not only the accumulation of storage lipids but also the precise coordination of membrane lipid remodeling, signaling lipid regulation, and metabolic adaptation. These findings provide a valuable lipidomic perspective for understanding the molecular mechanism of bovine adipogenesis.

## 1. Introduction

Adipogenesis, the process by which precursor cells differentiate into mature adipocytes, is fundamental to mammalian development, energy homeostasis, and metabolic health [1,2]. This complex process is characterized by significant morphological and biochemical alterations, including extensive remodeling of the cytoskeleton and extracellular matrix, activation of specific transcriptional cascades (e.g., involving peroxisome proliferator-activated receptor γ (PPARγ) and CCAAT/enhancer binding protein α (C/EBPα)), and, most notably, the massive accumulation of intracellular lipids [3,4]. Understanding the molecular mechanisms governing adipogenesis is crucial, not only for basic biology but also for addressing obesity-related metabolic disorders and improving livestock meat quality.

Bovine adipose-derived neural crest stem cells (baNCSCs) represent a unique and promising model for studying adipogenesis [5,6]. Our previous work successfully identified and isolated baNCSCs from the adipose tissue of Jinnan Cattle, one of China’s five major yellow cattle breeds [5]. Subsequently, transcriptomics techniques were employed to confirm that baNCSCs have the ability to differentiate into adipocytes [6]. These findings confirmed the existence of multipotent stem cells with neural crest characteristics in bovine adipose tissue [5,6]. These cells possess the capacity to differentiate into multiple lineages, including adipocytes, offering a valuable system to decipher the species-specific regulatory networks controlling fat deposition.

Although transcriptomic and proteomic studies have shed light on the gene expression changes during adipogenesis [6,7,8], the dynamic changes in the global lipidome, which represents the ultimate functional output of this process, remain less explored, especially in specific cellular models. Lipidomics, a comprehensive analysis of lipid molecules within a biological system, provides a powerful tool to uncover the intricate changes in lipid composition, abundance, and metabolism [9,10]. It can reveal how specific lipid classes and subclasses, such as glycerophospholipids (GP), sphingolipids (SP), and glycerides (GL), are reprogrammed to support the formation of lipid droplets, membrane expansion, and signal transduction during differentiation [10,11]. However, a detailed lipidomic profiling of adipogenic differentiation from baNCSCs is still lacking. How the entire lipidome evolves from the undifferentiated state to the terminally differentiated adipocyte state in this specific model is unknown.

Therefore, this study aimed to employ an ultra-high-performance liquid chromatography–mass spectrometry (UHPLC-MS/MS)-based lipidomics approach to (1) comprehensively identify and quantify the lipid profiles of baNCSCs at different stages of adipogenic differentiation (day 0 vs. day 9); (2) characterize the differentially abundant lipid species and subclasses that mark the progression of differentiation; and (3) elucidate the potential biological significance of these lipid metabolic shifts in driving adipogenesis. Our findings will provide a deep insight into the lipid dynamics of adipocyte differentiation and contribute to a more holistic understanding of fat development in bovines.

## 2. Materials and Methods

### 2.1. Cell Culture and Sample Collection

The baNCSCs cells were isolated and preserved by the laboratory of the College of Animal Science and Technology, Shanxi Agricultural University (Taiyuan, China). The baNCSCs (2.1 × 10^4^/cm^2^) that had been passed down to 5 generations (P5) were seeded in a 15 cm culture dish (NEST, Wuxi, China) and cultured in the growth medium [DMEM medium (High Glucose, Glutamax; Gibco, Carlsbad, CA, USA) supplemented with 10% fetal bovine serum (FBS; Gibco, Carlsbad, CA, USA), 1 mM sodium pyruvate (Sigma, Saint Louis, MO, USA), 50 mg/mL uridine (Sigma, Saint Louis, MO, USA), 50 units/mL pen strep (Gibco, Carlsbad, CA, USA), and 1.25 mg/mL amphotericin B (Gibco, Carlsbad, CA, USA)] at 37 °C in a 5% CO_2_ incubator. When the density of P6-generation cells in the culture dish reached 100% and contact inhibition was inhibited for 1 day, adipocyte induction differentiation was carried out using adipogenic differentiation medium [(α-MEM medium (Gibco, Carlsbad, CA, USA) supplemented with 1% adipogenic supplement (R&D, Minneapolis, MN, USA), 10% FBS (Gibco, Carlsbad, CA, USA), 50 units/mL pen strep (Gibco, Carlsbad, CA, USA), and 1.25 mg/mL amphotericin B (Gibco, Carlsbad, CA, USA)]. The adipogenic differentiation medium was changed once every 3 days. Cell samples were collected on the 0th day and the 9th day and set as the control group (CON0) and the 9-day differentiation group (DIF9), respectively. The experiments were performed three times.

### 2.2. Lipid Extraction

Samples of 5 × 10^6^ cells were added to a centrifuge tube, followed by the addition of 0.75 mL of pre-cooled methanol. The mixture was shaken for 30 min. Then, 2.5 mL of pre-cooled methyl tert-butyl ether was added and incubated at room temperature on a shaker for 1 h. Next, 700 μL of mass-spectrometry-grade ultrapure water was added and mixed thoroughly. The mixture was incubated at room temperature for 10 min, followed by centrifugation at 1000 rpm for 15 min. The upper hydrophobic phase was collected, and 1 mL of a mixed solution with a volume ratio of V (methyl tert-butyl ether):V (methanol):V (water) = 10:3:2.5 was added to the lower layer for extraction. The upper hydrophobic phase was collected again and concentrated using a nitrogen evaporator. The sample was dissolved in 100 μL of isopropyl alcohol, 75 μL of the lipid extract was taken, and it was introduced into the high-performance liquid chromatography–mass spectrometry system for detection and analysis. Equal amounts of lipid extracts were taken from the processed samples, mixed evenly as data quality control (QC) samples, and tested at intervals before, during, and after the samples to be tested were injected into the machine.

### 2.3. Chromatographic Conditions

The target compound was isolated using ultra-high-performance liquid chromatography (UHPLC) (VanquishTM UHPLC; Thermo, San Jose, CA, USA). The chromatographic conditions were as follows: mobile phase A consisted of 40% water, 60% acetonitrile, 0.1% formic acid, and 10 mmol/L ammonium acetate, while mobile phase B consisted of 10% acetonitrile, 90% isopropanol, 0.1% formic acid, and 10 mmol/L ammonium acetate. The flow rate of the mobile phase was 0.35 mL/min, the column temperature was set at 40 °C, the injection volume was 5 μL, and the chromatographic gradient elution procedure is outlined in Table S1.

### 2.4. Mass Spectrometry Conditions

The metabolites eluted from the chromatographic column were detected using the high-resolution mass spectrometer (Q ExactiveTMHF; Thermo, San Jose, CA, USA) in the information-dependent acquisition (IDA) mode. Each sample was collected in both the positive ion (POS) mode and the negative ion (NEG) mode. The conditions were as follows: sheath gas flow rate of 20 au, auxiliary gas flow rate of 5 au (7 au for negative ions), S-Lens RF level of 50, electrospray voltage of 3 kV, capillary temperature of 350 °C, mass-to-charge ratio (*m*/*z*) scanning range of 114–1700, and heater temperature of 400 °C. The automatic gain was set at 1 × 10^6^, with a dynamic exclusion of 15 s, standard collision energies of 25 and 30 ev (20, 24, and 28 ev for negative ions), S-Lens RF level of 50, injection time of 100 ms, and scanning of QC samples to correct experimental systematic errors based on their variations.

### 2.5. Data Processing and Statistical Analysis

The original data obtained from the analysis was imported into the Compound Discoverer 3.1 software. Screening and preprocessing were conducted using parameters such as retention time and mass-to-charge ratio. Peak alignment was performed for different samples with a retention time deviation of 0.2 min and a mass deviation of 5 ppm. Additional settings included a signal-to-noise ratio of 3, a minimum signal intensity of 100,000, and a signal intensity deviation of 30%. Chromatographic peaks in the samples were integrated to obtain relative quantitative values, and the quantitative results were standardized using the total peak area. This approach provides relative quantification, which allows for the comparison of abundance changes for each lipid species across samples but does not reflect absolute concentrations. The molecular weight of lipids was determined based on the mass-to-charge ratio, while the molecular formula was predicted by analyzing the deviation in the molecular mass number, ion peak, and fragment ion. The qualitative and quantitative results of lipid compounds were obtained by comparing annotations in the self-built libraries Lipidmaps and Lipiblast.

Principal component analysis (PCA) and partial least square discriminant analysis (PLS-DA) were performed on lipid compounds to analyze the sample clustering within the group of baNCSCs before and after differentiation and the separation trend between groups, as well as to interpret the relationships and differences between samples and between lipid compounds. The differential analysis of DIF9 vs CON0 was conducted. Differential lipid metabolites were screened using a combination of univariate statistical analysis of fold change (FC), *t*-test *p* value, and VIP of the PLS-DA model, with criteria of |log2(FC)| ≥ 1 (FC ≥ 2 or ≤0.5), VIP ≥ 1, and *p* < 0.05. The overall changes in the relative content of lipid subtypes in cells were calculated based on the peak areas of different lipid types.

## 3. Results

### 3.1. Sample Quality Control and Method Reliability Evaluation

**Figure 1:**
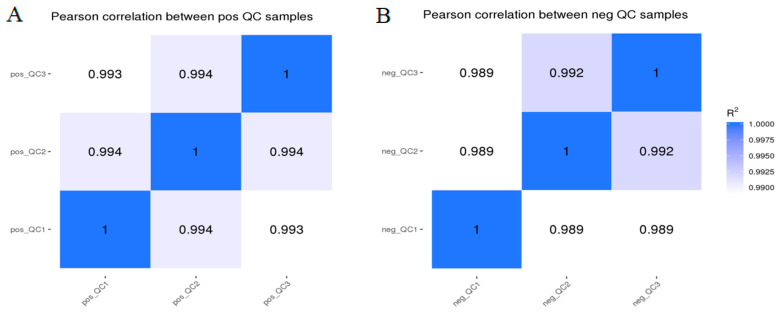
The correlation of QC sample in the positive (**A**) and negative (**B**) ion mode.

The Pearson correlation coefficients among the QC samples are shown in Figure 1. The R^2^ values in this study range from 0.989 to 0.994, indicating a high correlation among QC samples. The basic peak chromatograms of lipid extract from adipocytes of QC samples in the positive (POS) and negative (NEG) electrospray modes were observed. The total ion current chromatogram of the sample demonstrates good overlap in retention time and peak area, suggesting a stable detection process and high data quality (Figure S1).

### 3.2. Intracellular Lipid Identification

**Figure 2:**
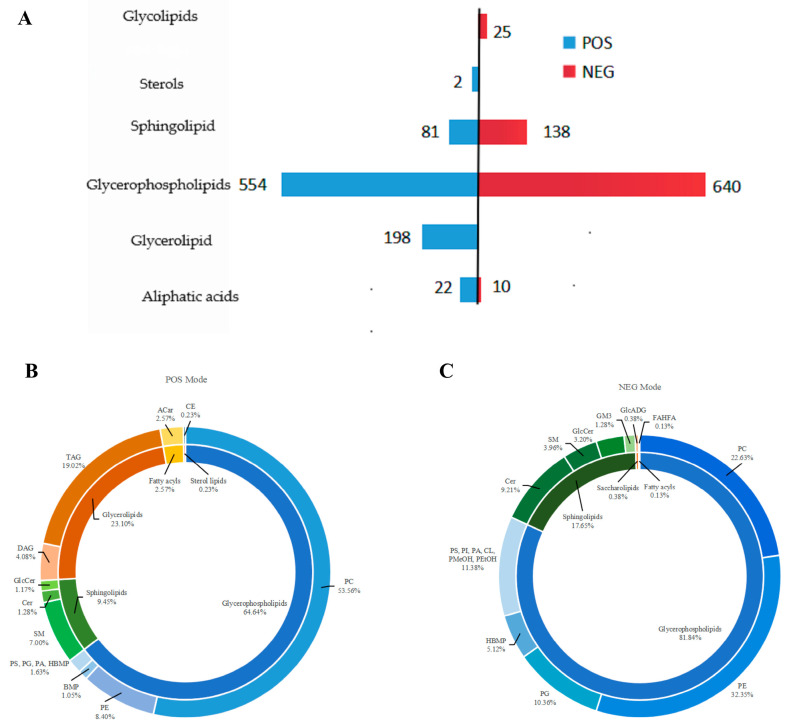
Intracellular lipid identification. (**A**) The number of lipid classes in cells under positive and negative ion modes; (**B**) percentages of numbers of lipid subclasses in positive ion mode; (**C**) percentages of numbers of lipid subclasses in negative ion mode. Acar: acyl carnitines; BMP: bis (monoacyl) glycerophosphate; CE: cholesterol esters; Cer: ceramides; CL: cardiolipins; DAG: diacylglycerols; FAHFA: hydroxyfatty acid lipid; GlcADG: glucuronic acid diacylglycerol; GlcCer: glucuronic ceramides; GM3: gangliosides; HBMP: hemibi (monoacyl) glycerophosphate; PA: phosphatidylic acid; PC: phosphatidylcholine; PE: phosphatidylethanolamine; PEtOH: phosphatidylethanol; PG: phosphatidylglycerol; PI: phosphatidylinositol; PMeOH: phosphatidylmethanol; PS: phosphatidylserine; SM: sphingomyelin; TAG: triacylglycerols.

The spectral fragment ions, collision energy, and other information of each lipid compound in the secondary spectra were matched and identified with the secondary mass spectra in the self-built libraries Lipidmaps and Lipidblast. Lipid compounds with a coefficient of variation of less than 30% in the QC samples were retained, resulting in the identification of a total of 1639 lipids, including 857 in POS modes and 782 in NEG modes (Figure 2A).

Under the POS mode, lipids consist of 22 types of aliphatic acids (FAs), 198 types of GL, 554 types of GP, 81 types of SP, and 2 types of sterols (ST) (Figure 2A). Among them, FAs include 22 types of acyl carnitines (ACar; 2.57%), GL include 35 types of diacylglycerols (DAG; 4.08%), and 163 types of triacylglycerols (TAG; 19.02%); GP include 459 types of phosphatidylcholine (PC; 53.56%), 72 types of phosphatidylethanolamine (PE; 8.40%), 3 types of phosphatidylserine (PS), 5 types of phosphatidylglycerol (PG), 2 types of phosphatidylic acid (PA), 4 types of hemibi (monoacyl) glycerophosphate (HBMP) (PS, PG, PA, and HBMP 1.63%), and 9 types of bis (monoacyl) glycerophosphate (BMP; 1.05%). SP include 60 types of sphingomyelin (SM; 7.00%), 11 types of ceramides (Cer; 1.28%), and 10 types of hexose ceramides (HexCer; 1.17%). ST identified two cholesterol esters (CE; 0.23%) (Figure 2B).

Under the NEG model, lipids comprise 640 types of GP, 138 types of SP, 10 types of FAs, and 25 types of glycolipids (SL) (Figure 2A). Among them, GP include 177 types of PC (22.63%), 253 types of PE (32.35%), 23 types of phosphatidylinositol (PI; 2.94%), 27 types of PS (3.45%), 4 types of PA (0.51%), 26 types of CL (3.32%), 1 type of phosphatidylmethanol (PMeOH; 0.38%), 8 types of phosphatidylethanol (PEtOH; 1.02%), 81 types of PG (10.36%), and 40 types of HBMP (5.12%); SP include 72 types of Cer (9.21%), 25 types of HexCer (3.20%), 31 types of SM (3.96%), and 10 types of gangliosides (GM3; 1.28%); FAs identified one type of hydroxyfatty acid lipid (FAHFA; 0.13%); SL comprise three types of glucuronic acid diacylglycerol (GlcADG; 0.38%) (Figure 2C).

### 3.3. Principal Component Analysis

**Figure 3:**
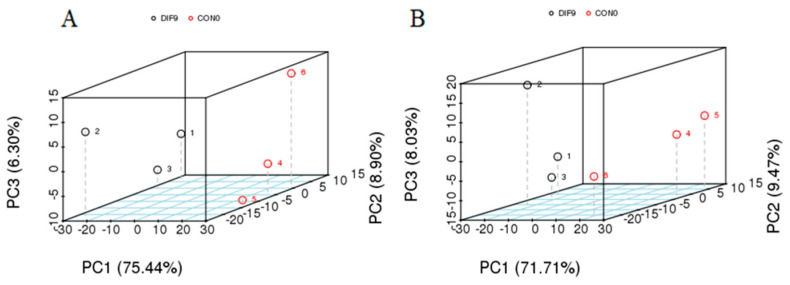
The PCA three-dimensional diagram in the (**A**) positive and (**B**) negative ion modes, respectively. Axes PC1, PC2, and PC3 represent the scores of the principal components ranked first, second, and third, respectively.

PCA can provide initial insights into the overall metabolic differences between groups and the variability within each group’s samples. Multidimensional data from the DIF9 and CON0 groups were collected for dimensionality reduction and regression analysis (Figure 3). It showed that, in the POS mode (Figure 3A), the variance coefficients for principal component 1, principal component 2, and principal component 3 are 75.44%, 8.90%, and 6.30%, respectively. In the NEG mode (Figure 3B), the variance coefficients for principal component 1, principal component 2, and principal component 3 are 71.71%, 9.47%, and 8.03%, respectively. The samples within the DIF9 and CON0 groups exhibit clustered distributions, showing a tendency of separation between the groups, suggesting differences in lipid profiles between DIF9 and CON0.

### 3.4. Partial Least Square Discriminant Analysis

**Figure 4:**
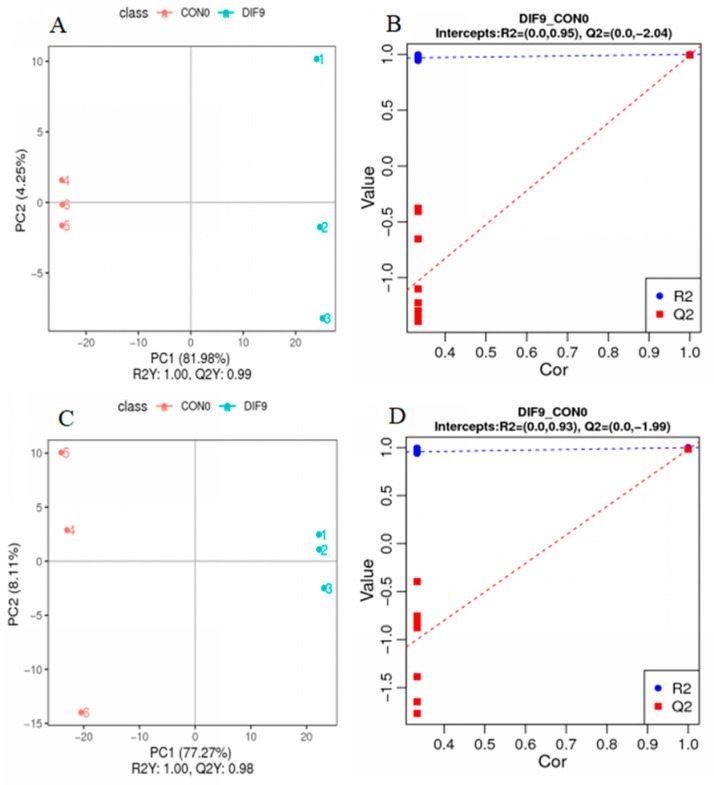
Scattered point chart and sequencing verification chart of PLS-DA for DIF9 and CON0. (**A**,**B**) PLS-DA score chart and sequencing verification chart under positive ion mode, respectively; (**C**,**D**) PLS-DA score chart and sequencing verification chart under negative ion mode, respectively.

The differences between groups were analyzed using PLS-DA. As shown in Figure 4, in both the POS (Figure 4A,B) and NEG modes (Figure 4C,D), the R2Y and Q2 values of the PLS-DA model were both close to 1, with stable values, indicating the stability and reliability of the model. The model was sorted and validated, revealing that when the R2 value exceeded the Q2 value and the intercept of the Q2 regression line with the Y-axis was less than 0, it suggested that the model was not overfitted, possessed high reliability and predictive capability, and could be further utilized for screening of differential metabolic markers.

### 3.5. Volcanic Map of Differential Lipid Compounds

**Figure 5:**
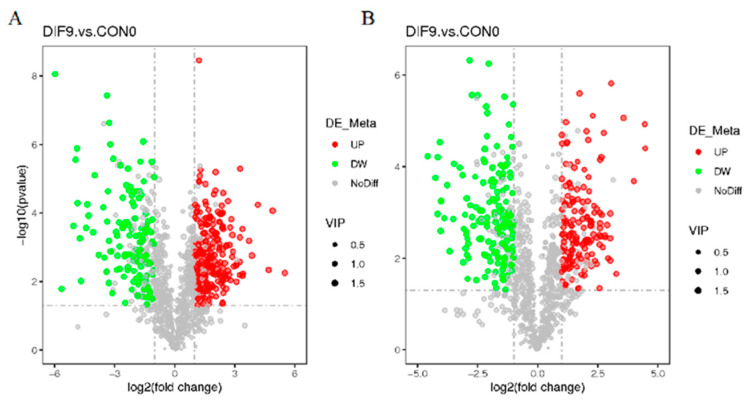
Volcanic map of differential lipid metabolites before and after adipocyte differentiation. (**A**,**B**) The volcano map under positive and negative ion modes, respectively. The horizontal coordinate represents the expression multiples of lipid compounds in different groups, the vertical coordinate represents the significance level of differences, and dots represent lipid compounds; red dots represent significantly upregulated lipids, green dots represent significantly downregulated lipids, gray dots represent insignificant differences, and the size of the dots represents the VIP value.

The volcano plot of differential lipid compounds can simultaneously display the information of the three dimensions of *p*-value, fold change, and VIP in the metabolites. It show that under the POS (Figure 5A) and NEG modes (Figure 5B), a considerable number of differential lipid metabolites between DIF9 and CON0 exhibit significant upward and downward adjustment trends.

### 3.6. Analysis of Differential Lipid Compounds

Using FC ≥ 2 or ≤0.5, VIP ≥ 1, and *p* < 0.05 as screening criteria, lipids meeting the threshold were identified as differential lipid compounds. Table 1 shows that under both the POS and NEG models, a total of 6 major categories, 20 subcategories, and 568 differential lipid metabolites were identified in the DIF9 group and the CON0 group. Among these, there were 105 types of GL, with 102 types showing an increased relative content (97.14%). Additionally, there were 358 types of GP, with 200 types exhibiting a relative increase in content (55.87%). Furthermore, there were 95 types of SP, with 77 types showing a relative decrease in content (80.21%). Seven types of FAs were identified, all of which had increased relative contents. Type SL showed a relative decrease in content, while two types of ST both displayed decreased relative contents. The changes in lipid content within each subclass were estimated by analyzing the peak area of lipids within the same subclass. Table 2 presents specific information on the differences in lipid subclass compounds between the DIF9 group and the CON0 group.

## 4. Discussion

Under the influence of adipogenic factors, NCSCs differentiate into mature adipocytes capable of fat storage, with lipids serving as fundamental metabolites and cellular biomarkers that reflect various biological states and cellular activities [12,13]. In the adipogenic-differentiated baNCSCs, the types and relative contents of TAG and DAG lipid metabolites were significantly increased compared with those before differentiation, confirming that the mature adipocytes have completed differentiation and achieved substantial lipid accumulation. TAG and DAG are glycerolipid substances that serve as marker lipid metabolites, which are induced to differentiate into mature adipocytes in vitro and accumulate throughout the adipogenic differentiation process [10]. This lipid accumulation is consistent with our transcriptomic findings [6], which showed significant enrichment of differentially expressed genes (DEGs) in fatty acid metabolism and glycerophospholipid metabolism pathways during the middle-to-late stages of differentiation (DIF9 vs. CON0), providing transcriptional support for the observed lipidomic changes. Adipogenesis is defined as the process by which adipose-derived stem cells differentiate into mature adipocytes, characterized by the emergence and aggregation of intracellular lipid droplets. The core of these lipid droplets is primarily composed of neutral lipids, predominantly represented by glyceride substances [14]. TAG and DAG are the main energy sources in animals, and the increase in glyceride metabolites during experimental differentiation highlights their crucial role in sustaining the organism’s fundamental physiological metabolic functions.

GP are the primary lipids found in cell membranes, playing crucial roles in regulating membrane transport, signal transduction, endocytosis, cell growth, and microdomain organization [15]. During adipocyte differentiation, the composition of GP undergoes significant changes to accommodate cellular functions [16]. In terms of GP, a comparison of the non-differentiated baNCSCs and the adipogenic-differentiated cells revealed an increase in the types of PE, PS, PA, CL, PG, and BMP in the differentiated cells. Conversely, the types of HBMP decreased, while the increases and decreases in phosphatidylcholine PC and phosphatidylinositol PI were nearly balanced. These results indicate that adipogenic differentiation significantly impacts the composition of the cell membrane, leading to membrane remodeling. The transcriptomic analysis of the same differentiation model revealed significant enrichment of DEGs in the phosphoinositide 3-kinase–serine/threonine kinase (PI3K-AKT) and mitogen-activated protein kinase (MAPK) signaling pathways, which are known to regulate membrane dynamics and cytoskeletal reorganization, providing a potential regulatory context for the observed lipid restructuring [6]. The presence of the sn1 vinyl ether bond in specific PE species is known to enhance membrane packing density and rigidity in vitro, and an increase in its concentration can significantly influence membrane physical properties [17]. However, this study lacks direct functional assays (e.g., membrane fluidity measurements) to substantiate the claim about membrane rigidity changes. The phospholipids of C20:4 in PE can promote fat differentiation and the formation of larger lipid droplets [18], which is consistent with the present study. PS is associated with energy storage in lipid droplets [19]. The increase in the types and abundance of PS is essential for the activation of signaling pathways and proteins, thereby facilitating the differentiation of adipocytes in NCSCs [20]. PC regulates the biological functions of nerve cells by influencing cell permeability, myelin formation, and mitochondrial activity [21]. Therefore, changes in PC levels are of great significance to NCSCs. Research indicates that PC, as a surfactant, promotes the maturation of primary lipid droplets by enhancing the cells’ ability to synthesize and store triglycerides (TG), with its content increasing alongside fat accumulation [22]. This study observed that the levels of certain PC subclasses increased while others decreased, indicating that different PC species may exert distinct regulatory effects on lipid droplet formation. Notably, the PC subclasses exhibiting significant alterations following differentiation, including PC (16:2/22:6), PC (16:2/20:5), and PC (14:0/16:3), consistently demonstrated an upward trend in their relative abundance. Furthermore, the size of the PC/PE ratio is negatively correlated with cell membrane permeability, which activates inflammatory-factor-mediated liver injury [23]. Therefore, in the present study, the increase in certain PC contents can be sustained by regulating the PC/PE ratio to preserve the normal function of the cell membrane. PA is a key component in the synthesis of membrane phospholipids and TG, reflecting the capacity for membrane phospholipid production necessary for cell mass doubling [24]. An increase in intracellular PA levels can result in the formation of larger lipid droplets [25], which is consistent with the findings observed in this study. CL is primarily located in the inner mitochondrial membrane, facilitates reverse signaling from mitochondria to the nucleus, enhances the transport of mitochondrial components, and plays a crucial role in the positive regulation of the thermogenic function of adipocytes [26]. The present study found that the types and relative amounts of CL increased, indicating an accelerated synthesis of CL in the mitochondria of adipocytes, which, in turn, promotes fat heat generation and glucose uptake, thereby exerting a physiological regulatory role of adipocytes in the body. PI generates derivatives through phosphorylation and plays a crucial role in signal transduction, the exchange of materials across the membrane, and the regulation of the actin cytoskeletal network, which is essential for maintaining and modulating cell morphological changes [27]. Therefore, it was observed that the types of PI changed relatively stably in the present study.

In terms of SP, during the adipogenic differentiation of baNCSCs, the types and relative contents of Cer and SM lipid subclasses within the cells both decreased. Meanwhile, the HexCer lipid subclass exhibited two distinct changes: an increase and a decrease. These observations suggest that the distribution patterns of SP differ before and after adipogenic differentiation. This lipidomic finding aligns with the transcriptomic data [6], which showed downregulation of key hub genes like HRAS and PTEN in the middle/late differentiation stage (DIF9 vs. CON0). These genes are important regulators within the enriched PI3K-AKT signaling pathway [6], suggesting a potential link between decreased sphingolipid levels and altered activity in this critical adipogenic pathway. The primary SM in bovine cells is SM, which serves as a crucial signaling lipid in the cell membrane, facilitating the transmission of essential cellular signals and playing a significant role in regulating processes such as cell growth, differentiation, and apoptosis while also maintaining a balance between the stimulation and inhibition of lipogenesis [28]. Cer can be generated by hydrolyzing SM with sphingolipase [29], while SM is primarily synthesized from Cer through sphingomyelin synthase [30]. This indicates that Cer serves as both a precursor for SM synthesis and a decomposition product of SM. Therefore, in this study, Cer exhibits a similar trend during the adipogenic differentiation process of baNCSCs. HexCer is a glycosphingolipid formed by the linkage of sugar to the ceramide portion via fatty acyl groups, serving as a crucial component of the cell surface and playing a significant role in the differentiation of various types of adipose tissue [24,31]. However, due to the structural complexity and diversity of cellular glycolipids, along with the variations in abundance, chemical stability, and biophysical properties among different analytes, there are currently limited research reports available. In the present study, different lipid subclasses of SP showed varying content changes, suggesting that each subclass may play distinct regulatory roles during cell differentiation.

In terms of FAs, with the progress of adipocyte differentiation of baNCSCs, the types and relative contents of FAHFA and ACar in the cells increased, while no significant changes were observed in intracellular free fatty acids (FFA). This indicates that adipocyte differentiation adapts to and maintains the changes in the physiological metabolic state of the cells by stabilizing the content of FAs. The branched-chain FAHFA within the FAHFA classification plays potential roles in metabolism and inflammatory regulation. Esterification onto the glycerol backbone can lead to the formation of TG containing FAHFA [32]. A previous study by Yore et al. [33] showed that FAHFA was elevated in adipocytes of mice with overexpression of GLUT-4, which was consistent with the results of this study. Adipocytes can regulate adipocyte insulin sensitivity and glucose tolerance by increasing FAHFA content [34]. ACar is a metabolite of carnitine and acyl coenzymes, responsible for transporting fatty acids to the mitochondrial matrix for fatty acid β-oxidation, which contribute to heat production in adipose tissue [35]. The increase in the relative content of ACar in carbon chains of different lengths in the present study indicates that mature adipocytes regard ACar as an important substrate for lipid oxidation and exert their physiological metabolic regulatory functions. The increase in the relative content of ACar in carbon chains of different lengths in the present study indicates that mature adipocytes regard ACar as an important substrate for lipid oxidation. This finding is supported by the transcriptomic data [6], which showed upregulation of genes involved in fatty acid oxidation pathways, suggesting enhanced β-oxidation capacity in differentiating adipocytes. FFA serves as the energy source and precursor for various bioactive lipid molecules and membrane lipids, with the carbon chain length, degree of unsaturation, and position of double bonds of fatty acids all influencing the formation of fats [36]. Unsaturated FFA can act as endogenous ligands that directly bind to and activate the expression levels of lipid-related genes [37]. A study conducted by Kim [38] also suggests that high levels of FFA are a sign of white adipocyte dilation, low energy expenditure, or β-oxidation disorder. Therefore, the FFA observed in the present study remained unchanged, indicating that the cell state after adipocyte induction was favorable.

In terms of ST, as baNCSCs differentiate into adipocytes, the types and relative contents of CE within the cells decrease, indicating that the reduction in CE content may enhance the adipogenic differentiation of baNCSCs. CE is a crucial lipid compound of ST, influencing the fluidity of membrane lipids, which plays a vital role in cellular physiology, particularly in processes such as cell proliferation and differentiation [39]. Therefore, CE is an effective and important factor in reducing fat production [40]. The present study showed that the content of CE decreases in the later stage of differentiation, which can increase the fluidity of the cell membrane and enhance the sensitivity to growth factors, and this is one of the effects promoting the regulatory process of cell differentiation.

The overall results of the experiment indicated that the types and relative contents of lipids containing unsaturated fatty acid structural components, such as oleic acid (18:1) and palmitoleic acid (16:1), increased, suggesting that enzyme activity on the cell membrane surface and signal transduction were enhanced during the differentiation process of baNCSCs. This is supported by the transcriptomic analysis [6], which identified upregulation of key hub genes like epidermal growth factor receptor (EGFR) and MYC during differentiation (DIF9 vs. CON0), genes known to influence signal transduction and metabolic reprogramming. These unsaturated fatty acids are the main monounsaturated fatty acids that constitute GP, GL, and CE, and the ratio of them to saturated fatty acids plays a crucial role in the structure and function of cell membranes [41]. The content of unsaturated fatty acid chains in the phospholipid bilayer is positively correlated with the fluidity of the cell membrane [42]. Therefore, baNCSCs enhance their cellular activity during adipogenic differentiation by increasing the proportion of unsaturated fatty acids in their lipid composition. During the adipocyte differentiation process of NCSCs, there are many types of lipids with significant changes in their relative contents, such as the glycolipid macromolecular substance GlcADG, which is usually studied more in plants [43]. Phospholipids BMP and HBMP have not been specifically described in the literature yet, and their mechanisms of influencing adipogenic differentiation still require further research. Different in vitro adipogenesis and differentiation models reflect distinct characteristics of adipose tissue [44]. The present study systematically investigated the composition of and alterations to intracellular lipid profiles during the differentiation process of adipocytes induced by baNCSCs. It also examined the roles of key lipid subclasses and differential lipid metabolites in adipogenic differentiation. This study reveals the correlation between the composition of and changes in specific lipids and the adipogenic differentiation of baNCSCs, thereby enhancing the understanding of the lipid molecular metabolic kinetics involved in the differentiation mechanism of adipocytes.

Although this study provided comprehensive lipidomics analysis results, there are still certain limitations that need to be acknowledged. The experimental model in the present study was limited to in vitro cell culture and lacked in vivo validation, such as in animal models, which means that the physiological relevance of the observed lipid dynamics in the context of whole-body metabolism and actual fat deposition in cattle requires further investigation. Additionally, the study identified several lipid subclasses (e.g., BMP, HBMP, GlcADG) with significant changes but unclear biological functions in adipogenesis. Further functional studies, such as genetic knockdown or overexpression of enzymes involved in their metabolism, are needed to clarify their specific roles. Addressing these limitations in future work will deepen the mechanistic understanding of adipogenesis in baNCSCs and enhance the practical implications for improving livestock traits.

## 5. Conclusions

This lipidomics study reveals that adipogenic differentiation of baNCSCs is driven by three fundamental lipid metabolic shifts: (1) Significant accumulation of TAGs and DAGs confirms successful maturation into lipid-storing adipocytes. (2) Comprehensive restructuring of glycerophospholipids (e.g., PE, PS, PA, CL) facilitates membrane expansion and supports enhanced signaling capabilities required for differentiation. (3) Contrasting changes in sphingolipids (decreased Cer/SM) versus specialized fatty acid derivatives (increased FAHFA/ACar) fine-tune the adipogenic process. These descriptive findings provide a foundation for understanding lipid metabolism in bovine adipogenesis, offering insights for future research on livestock metabolic biology.

## Figures and Tables

**Table 1:** Variation ratio of lipid subclass metabolites in adipocyte differentiation.

Lipid Categories	Lipid Subclasses	Up Numbers/Percentage	Down Numbers/Percentage	Total Changes
Glycerides	DAG	11/91.67%	1/8.33%	12
	TAG	91/97.85%	2/2.15%	93
Glycerophospholipids	PA	2/100%	0/0	2
	PC	83/47.98%	90/52.02%	173
	PE	54/62.79%	32/37.21%	86
	PG	21/53.85%	18/46.15%	39
	PS	5/100%	0/0	5
	PI	4/50%	4/50%	8
	CL	16/100%	0/0	16
	PEtOH	0/0%	1/100%	1
	BMP	6/100%	0/0	6
	HBMP	9/40.91%	13/59.09%	22
Sphingolipids	HexCer	10/83.33%	2/16.67%	12
	Cer	4/16%	21/84%	25
	SM	1/1.85%	53/98.15%	54
	GM3	3/75%	1/25%	4
Aliphatic acids	FAHFA	1/100%	0/0	1
	ACar	6/100%	0/0	6
Lycolipids	GlcADG	0/0	1/100%	1
Sterols	CE	0/0	2/100%	2

Acar: acyl carnitines; BMP: bis (monoacyl) glycerophosphate; CE: cholesterol esters; Cer: ceramides; CL: cardiolipins; DAG: diacylglycerols; FAHFA: hydroxyfatty acid lipid; GlcADG: glucuronic acid diacylglycerol; GM3: gangliosides; HBMP: hemibi (monoacyl) glycerophosphate; HexCer: hexose ceramides; PA: phosphatidylic acid; PC: phosphatidylcholine; PE: phosphatidylethanolamine; PEtOH: phosphatidylethanol; PG: phosphatidylglycerol; PI: phosphatidylinositol; PS: phosphatidylserine; SM: sphingomyelin; TAG: triacylglycerols.

**Table 2:** Composition and changes in lipid metabolites in adipocyte differentiation.

Lipid Categories	Lipid Subclass Metabolites	Ion Mode	FC	Up/ Down	VIP
GL	DAG (16:1)/18:3)	pos	5.88	up	1.12
	DAG (16:1/22:6)	pos	5.24	up	1.25
	DAG (14:0/18:3)	pos	3.74	up	1.28
	1-O-(1Z-Tetradecenyl)-2-(9Z-octadecenoyl)-sn-glycerol	pos	3.3	up	1.46
	DAG (18:0/22:4)	pos	3.09	up	1.51
	TAG (20:2-22:5-22:6)	pos	7.73	up	1.51
	TAG (18:1-22:5-22:5)	pos	7.36	up	1.25
	TAG (22:1-22:4-22:5)	pos	7.29	up	1.42
	TAG (22:1-22:5-22:5)	pos	7.08	up	1.43
	TAG (16:1-22:5-22:6)	pos	6.03	up	1.51
GP	PA (16:1/18:1)	neg	2.35	up	1.25
	1-arachidoyl-sn-glycero-3-phosphate	pos	2.01	up	1.4
	PC (16:2/22:6)	pos	45.08	up	1.43
	PC (16:2/20:5)	pos	29.68	up	1.53
	PC (14:0/16:3)	pos	25.78	up	1.49
	PC (17:2/17:2)	pos	16.88	up	1.49
	OxPC (18:0-20:3+1O(1Cyc))	neg	16.79	up	1.49
	PE (20:5/22:6)	pos	9.57	up	1.47
	PE (16:1e/16:2)	neg	8.34	up	1.21
	PE (16:1e/16:0)	neg	8.11	up	1.54
	2-linoleoyl-sn-glycero-3-phosphoethanolamine	neg	7.61	up	1.25
	PE (16:0/18:2)	neg	6.73	up	1.34
	PG (20:5/20:5)	neg	8.1	up	1.56
	PG (18:1/19:1)	neg	0.13	down	1.6
	PG (14:0/16:0)	neg	0.15	down	1.44
	1,2-dioleoyl-sn-glycero-3-phospho-(1′-sn-glycerol)	pos	6.04	up	1.25
	PG (16:1(9Z)/20:4(5Z,8Z,11Z,14Z))	neg	5.5	up	1.43
	PS (18:0/22:4)	neg	3.04	up	1.07
	1-palmitoyl-2-stearoyl-sn-glycero-3-phosphoserine	neg	2.18	up	1.13
	PS (16:1(9Z)/18:1(9Z))	pos	2.31	up	1.25
	1-stearoyl-2-oleoyl-sn-glycero-3-phosphoserine	neg	2.25	up	1.16
	PS (18:0/22:4)	pos	2.22	up	1.22
	PI (18:1/20:5)	neg	9.65	up	1.18
	PI (16:1/20:4)	neg	5.2	up	1.15
	PI (18:0/20:2)	neg	0.19	down	1.52
	PI (17:0/20:4)	neg	0.22	down	1.31
	PI (16:0/22:6)	neg	0.29	down	1.44
	CL (16:0-18:2-16:1-18:1)	neg	6.97	up	1.08
	CL (16:1-18:1-16:1-18:2)	neg	5.11	up	1.04
	CL (16:1-18:1-18:2-18:2)	neg	5.04	up	1.04
	CL (14:0-18:2-16:1-18:1)	neg	4.63	up	1.26
	CL (16:1-18:1-18:2-20:4)	neg	4.15	up	1.03
	PEtOH (18:0-20:4)	neg	0.47	down	1.5
	BMP (22:6/22:6)	pos	6.56	up	1.53
	BMP (22:4/22:6)	pos	6.31	up	1.52
	BMP (22:5/22:5)	pos	5.24	up	1.54
	BMP (20:4/22:5)	pos	4.12	up	1.5
	BMP (20:4/22:6)	pos	2.95	up	1.51
	HBMP (22:5-22:6-16:1)	neg	11.9	up	1.58
	HBMP (18:0-20:1-18:1)	neg	0.11	down	1.4
	HBMP (18:0-18:1-18:0)	neg	0.13	down	1.12
	HBMP (16:0-22:6-16:0)	neg	0.13	down	1.14
	HBMP (18:0-18:1-16:0)	neg	0.14	down	1.3
SP	HexCer-NDS (d18:0/16:0)	neg	0.05	down	1.27
	HexCer-NS (d18:2/24:1)	pos	4.12	up	1.55
	HexCer-NS (d30:2/14:1)	neg	0.3	down	1.5
	HexCer-NS (d18:1/18:0)	pos	2.89	up	1.03
	HexCer-NS (d18:1/24:1)	neg	2.62	up	1.49
	Cer-NDS (d18:0/20:0)	neg	0.04	down	1.29
	Cer-NDS (d18:0/18:0)	neg	0.06	down	1.51
	Cer-NDS (d18:0/22:0)	neg	0.06	down	1.15
	Cer-NDS (d18:0/14:0)	neg	0.08	down	1.51
	Cer-NDS (d18:0/15:0)	neg	0.09	down	1.36
	N-[(15Z)-tetracosenoyl]sphinganine-1-phosphocholine	pos	0.02	down	1.22
	SM(d18:0/14:0)	neg	0.06	down	1.42
	SM (d14:0/18:0)	neg	0.07	down	1.42
	SM (d26:0/12:0)	pos	0.08	down	1.17
	SM (d14:0/22:0)	neg	0.09	down	1.34
	GM3 d34:0	neg	0.25	down	1.04
	GM3 d42:3	neg	2.76	up	1.15
	GM3 d42:1	neg	2.73	up	1.4
	GM3 d42:2	neg	2.22	up	1.21
FAs	FAHFA (22:6/22:5)	neg	7.39	up	1.59
	ACar 24:1	pos	9.63	up	1.39
	ACar 22:1	pos	6.92	up	1.21
	ACar 20:2	pos	3.85	up	1.35
	ACar 16:2	pos	3.57	up	1.04
	ACar 20:1	pos	2.96	up	1.32
SL	GlcADG (18:2-20:3)	neg	0.3	down	1.45
ST	CE 18:1	pos	0.03	down	1.35
	CE 20:3	pos	0.04	down	1.32

Note: The lipid compounds with the top 5 FC in each lipid subclass are listed and with fewer than 5 listed based on actual conditions. Acar: acyl carnitines; CE: cholesterol esters; Cer: ceramides; G cardiolipins; DAG: diacylglycerols; FAHFA: hydroxyfatty acid lipid; FAs: aliphatic acids; GL: glycerides; GlcADG: glucuronic acid diacylglycerol; GM3: gangliosides; GP: glycerophospholipids; HBMP: hemibi (monoacyl) glycerophosphate; HexCer: hexose ceramides; PA: phosphatidylic acid; PC: phosphatidylcholine; PE: phosphatidylethanolamine; PG: phosphatidylglycerol; PI: phosphatidylinositol; PS: phosphatidylserine; SL: glycolipids; SM: sphingomyelin; SP: sphingolipids; ST: sterols; TAG: triacylglycerols.

## Data Availability

The original contributions presented in this study are included in the article/Supplementary Materials. Further inquiries can be directed to the corresponding authors.

## Supplementary Materials

The following supporting information can be downloaded at: https://www.mdpi.com/article/10.3390/ani15213191/s1, Table S1: Chromatographic gradient elution procedure; Figure S1: The TIC of QC sample in the (A) positive ion mode and (B) negative ion mode.

## Abbreviations

The following abbreviations are used in this manuscript:

ACar	acyl carnitines
AKT	serine/threonine kinase
baNCSC	bovine adipose-derived neural crest stem cells
BMP	bis (monoacyl) glycerophosphate
CE	cholesterol esters
C/EBPα	CCAAT/enhancer binding protein α
Cer	ceramides
CHGs	core hub genes
CL	cardiolipin
DAG	diacylglycerols
DEGs	differentially expressed genes
EGFR	epidermal growth factor receptor
FAHFA	hydroxyfatty acid lipid
FAs	aliphatic acids
FBS	fetal bovine serum
FC	fold change
GL	glycerides
GlcADG	glucuronic acid diacylglycerol
GM3	gangliosides
GP	glycerophospholipids
HBMP	hemibi (monoacyl) glycerophosphate
HexCer	hexose ceramides
IDA	information-dependent acquisition
MAPK	mitogen-activated protein kinase
PA	phosphatidylic acid
PC	phosphatidylcholine
PCA	principal component analysis
PE	phosphatidylethanolamine
PEtOH	phosphatidylethanol
PG	phosphatidylglycerol
PI	phosphatidylinositol
PLS-DA	partial least square discriminant analysis
PMeOH	phosphatidylmethanol
PI3K	phosphoinositide 3-kinase
PPARγ	peroxisome proliferator-activated receptor γ
PS	phosphatidylserine
QC	quality control
SM	sphingomyelin
SP	sphingolipids
ST	sterols
TAG	triacylglycerols
UHPLC	ultra-high-performance liquid chromatography
VIP	variable importance in the projection
