# Supplementary material for: Lipidomics Techniques Revealed the Adipogenic Differentiation Mechanism of Bovine Adipose-Derived Neural Crest Stem Cells
Source: Animals (Basel). 2025 Nov 2;15(21):3191. doi: 10.3390/ani15213191 (PMC12606755; doi:10.3390/ani15213191)
Supplement: Supplementary file 1 [file animals-15-03191-s001.zip › animals-3949141-supplementary.pdf]

Table S1. Chromatographic gradient elution procedure

Time（min）	A Phase %	B Phase %
0	70	30
2	70	30
5	57	43
5.1	45	55
11	30	70
16	1	99
18	1	99
18.1	70	30
20	70	30

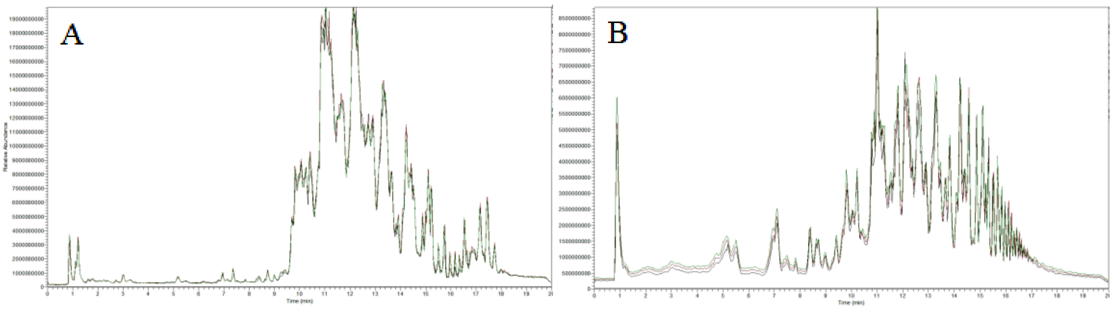

Figure S1. The TIC of QC sample in the positive ion mode(A) and negtive ion mode(B)
